# Association of *IL6* and *IL10* gene promotor polymorphisms with susceptibility to acute necrotizing encephalopathy

**DOI:** 10.3389/fnins.2023.1231957

**Published:** 2023-08-03

**Authors:** Ai Hoshino, Naoto Takahashi, Akira Oka, Masashi Mizuguchi

**Affiliations:** ^1^Department of Developmental Medical Sciences, Graduate School of Medicine, The University of Tokyo, Tokyo, Japan; ^2^Department of Pediatrics, The University of Tokyo Hospital, Tokyo, Japan; ^3^Department of Neuropediatrics, Tokyo Metropolitan Neurological Hospital, Fuchu, Japan; ^4^Department of Neurology, Saitama Children's Medical Center, Saitama, Japan; ^5^Department of Pediatrics, National Rehabilitation Center for Children with Disabilities, Tokyo, Japan

**Keywords:** acute necrotizing encephalopathy, cytokine storm, gene polymorphisms, susceptibility gene, case-control association analysis

## Abstract

**Objective:**

Acute necrotizing encephalopathy (ANE) is a severe complication of infectious diseases affecting the brain and systemic organs. The main pathogenesis is cytokine storm, in which interleukin-6 (IL-6) and interleukin-10 (IL-10) are candidates for key cytokines. To further elucidate their roles in the etiology and pathogenesis of ANE, we studied polymorphisms in the promotor regions of the *IL6* and *IL10* genes by genetic and functional analyses.

**Methods:**

We first conducted a case–control association study of four *IL6* and three *IL10* polymorphisms. We genotyped 31 Japanese ANE cases and compared the results with those of approximately 200 Japanese controls. For the two polymorphisms showing a possible association, we next studied whether the polymorphisms alter the production of IL-6 or IL-10 by lymphoblasts upon phorbol 12-myristate 13-acetate (PMA) stimulation.

**Results:**

The frequencies of *IL6* rs1800796G allele and *IL10* rs1800871/rs1800872 CC/CC diplotype were significantly higher in ANE cases than in controls. The *IL10* CC/CC diplotype was associated with low IL-10 production, whereas the *IL6* GG genotype was not associated with IL-6 production.

**Conclusion:**

*IL10* rs1800871/rs1800872 CC/CC diplotype may predispose Japanese children to ANE by altering IL-10 production in the early phase of infection. Etio-pathogenetic significance of *IL6* rs1800796G remains to be elucidated.

## 1. Introduction

Acute necrotizing encephalopathy (ANE) is a rare and fulminant syndrome of acute infection-triggered encephalopathy characterized pathologically by bilateral symmetric lesions in the thalamus and/or brainstem of vasogenic brain edema/necrosis (Mizuguchi et al., 1995). Antecedent infections of ANE are common viral diseases with high fever, such as influenza, exanthem subitum, rotavirus gastroenteritis and COVID-19 (Hoshino et al., 2012; Kasai et al., 2020; Sakuma et al., 2023). Being most common in childhood, ANE is an important cause of children’s deaths and severe neurological handicaps, in particular during influenza seasons.

ANE is a complex disorder in which multiple genetic and environmental factors are involved (Mizuguchi et al., 2023). ANE1, a familial and recurrent form of ANE prevalent in Caucasian ethnicities, is transmitted in an autosomal dominant fashion. Genetic studies have identified missense, loss-of-function mutations of the *RANBP2* gene as the main cause of ANE1 (Neilson et al., 2009). On the other hand, sporadic and non-recurrent ANE is most common in east Asian ethnicities. As a part of genetic susceptibility to sporadic ANE, a study in Japan have identified specific human leukocyte antigen (*HLA*) genotypes, DRB1*09:01 and DQB1*03:03 (Hoshino et al., 2016). However, much remains to be elucidated regarding the genetic susceptibility of ANE.

Despite the term “encephalopathy,” clinical course of ANE is characterized by profound systemic inflammation and multiorgan dysfunction: high fever and damages to the liver, kidney and muscle in most cases, as well as shock, disseminated intravascular coagulation and multiorgan failure in very severe cases. All these findings are consistent with “cytokine storm syndrome” (Mizuguchi et al., 2007). Previous studies have measured the levels of cytokines and other biomarkers in the blood and/or cerebrospinal fluid of sporadic ANE cases. Of the variable findings noted by these studies, an increase of serum interleukin-6 (IL-6) and that of serum interleukin-10 (IL-10) are very common, early and remarkable changes (Ichiyama et al., 2003; Akiyoshi et al., 2006; Mizuguchi et al., 2007; Kansagra and Gallentine, 2011; Kawamura et al., 2013; Okajima et al., 2022), suggesting important roles of these two cytokines in the pathophysiology of ANE.

To further explore the genetic background of ANE, we herein conducted a case–control study of single nucleotide polymorphisms (SNPs) in the promotor regions of the *IL6* and *IL10* gene that may alter the expression of these cytokines. For the candidate susceptibility variants obtained by the genetic studies, we did a functional analysis of their effects on cytokine production.

## 2. Materials and methods

### 2.1. Patients and controls

We recruited patients with ANE from hospitals throughout Japan from 2008 to 2019 based on the diagnostic criteria consisting of clinical course and characteristic MRI finding of symmetric brain lesions in the bilateral thalamus (Hoshino et al., 2012). Thirty-one patients, 15 males and 16 females aged from 8 months to 9 years and 7 months (median: 2 years and 2 months), were enrolled in the study. All patients were Japanese and mutually unrelated. None of them had a pathogenic mutation in the *RANBP2* gene. We obtained written informed consent from all guardians of patients participating in the study. This study was approved by the Ethics Committee of the Graduate School of Medicine, the University of Tokyo (No. G-3504).

We analyzed the *IL6* and *IL10* genotypes of control subjects, consisting of 100 healthy Japanese adults, 50 males and 50 females, 20 to 69 years of age, using DNA extracted from Pharma SNP Consortium B cell lines (PSC B cell lines) supplied by the Human Science Research Resources Bank (Osaka, Japan). We used the combined data of 100 Japanese controls from the Pharma SNP consortium and HapMap SNPs from the Japanese (JPT) Population database in International HapMap project for the variation frequencies of SNPs in *IL6* and *IL10* promoter regions.

### 2.2. Genotyping of *IL6* and *IL10* polymorphisms

Peripheral blood samples were collected from the patients. Genomic DNA was extracted from the blood samples using standard protocols. We analyzed four SNPs in the *IL6* promoter regions, rs1800797, rs1800796, rs2069829 and rs1800795, and three SNPs in the *IL10* promoter regions, rs1800896, rs1800871 and rs1800872. Two of them, rs1800871 and rs1800872, were in complete linkage disequilibrium in Japanese. Polymerase chain reaction (PCR) amplification of each *IL6* and *IL10* promoter region including the seven SNPs was performed using AmpliTaq PCR kits (Applied Biosystems). The reaction mixture contained 2 μL buffer, 2 μL of 2 mM dNTP, 1 μL forward and reverse primers (10 pmol), 0.12 μL AmpliTaq and 1 μL genomic DNA (30 ng). Primer sequences were the same as those used in a previous study (Chou et al., 2010) (Table 1). All the PCR products were purified with a PCR product sequencing kit (Amersham Biosciences, Little Chalfont, Buckinghamshire, United Kingdom), and were reacted with the Big Dye Terminator FS Ready Reaction kit (Applied Biosystems, Foster City, CA, United States). Purified PCR products were sequenced on 310 Genetic Analyzer, 3,100 Genetic Analyzer, or 3130xl Genetic Analyzer (Life Technologies, Carlsbad, CA, United States).

**Table 1:** Primer sequences.

Gene	SNP	Primer sequences (5′ to 3′)	Product size (bp)
***IL6***			
**Promoter − 597 G > A** **promoter -572 C > G**	**rs1800797** **rs1800796**	F: GCAAAGTCCTCACTGGGAGGA R: TCTGACTCCATCGGAGCCC	296
**Promoter − 190 C > T** **Promoter − 174 G > C**	**rs2069829** **rs1800795**	F: TGACTTCAGCTTTACTCTTTGT R: CTGATTGGAAACCTTATTAAG	188
***IL10***			
**Promoter − 1,082 A > G**	**rs1800896**	F: TTTCCAGATATCTGAAGAAGTCCTG R: GTAAGCTTCTGTGGCTGGAGT F-2: ATCCAAGACAACACTACTAAG	315
**Promoter − 819 T > C**	**rs1800871**	F: AGGCCAATTTAATCCAAGGT R: GTGCTCACCATGACCCCTAC	168
**Promoter − 627 A > C**	**rs1800872**	F: CCTAGGTCACAGTGACGTGG R: GGTGAGCACTACCTGACTAGC	412

F, forward primer; R, reverse primer; F-2, sequence primer.

### 2.3. Quantification of IL-6 and IL-10 production

Lymphoblasts from Japanese healthy controls (PSC B cell lines) were genotyped to identify three SNPs of *IL6* rs1800796, *IL10* rs1800871 and *IL10* rs1800872. Thirty-two lymphoblast samples were used for IL-6 quantification analysis and classified into three genotype groups, CC, CG and GG, of *IL6* rs1800796. The other 25 lymphoblast samples were used for IL-10 quantification analysis and classified into three diplotype groups, TA/TA, TA/CC and CC/CC, of *IL10* rs1800871/rs1800872.

Lymphoblastoid cells were cultured in RPMI 1640 medium containing inactivated 10% fetal bovine serum. Cell lines were dispensed into 6 well-cell plates with a constant cell count of 2.5 × 10^5^ cells/well. The three *IL6* genotypes and three *IL10* diplotypes were cultured in two wells per cell line with or without adding phorbol 12-myristate 13-acetate (PMA), a potent stimulant for lymphocytes mimicking inflammation. PMA stimulation was performed at 20 ng/mL and the conditions were adopted from a previous study (Yokoi et al., 1990). Cell plate was cultured for 48 h in 5% CO_2_ incubator. The ratio of IL-6 and IL-10 production in secretory supernatants from lymphoblast cell lines with and without PMA stimulation was analyzed among the three *IL6* genotype or *IL10* diplotype groups. IL-6 and IL-10 assays and quantification analysis were measured by using Bio-Plex™ Assay Kits and Bio-Plex™ system (Bio-Rad). Cytokine data analysis was performed using Bio-Plex Manager Software, ver. 6.1, following the procedure in the instruction manual of Bio-Plex™ Suspension Array System (171-00201JA).

### 2.4. Statistical analysis

We conducted a case–control association study of SNPs in the *IL6* and *IL10* promoter region using Chi-square test or Fisher’s exact test. The statistical analysis was conducted using Bell Curve for Excel (ver. 3.21; Social Survey Research information, Tokyo, Japan). The corrected *p*-values (*Pc*) were obtained by multiplying the uncorrected *p*-value with the number of comparisons, according to Bonferroni’s methods. A *Pc*-value <0.05 was considered as statistically significant. For quantification of IL-6 and IL-10 levels, the Steel-Dwass test was used, and a *p*-value <0.05 was considered as statistically significant.

## 3. Results

### 3.1. *IL6* and *IL10* gene polymorphisms

Genotyping of *IL6* polymorphisms revealed that the frequency of rs1800796 GG genotype was higher in ANE cases (9.7%) than in control subjects (4.2%). The frequency of G allele was significantly higher in ANE cases (37.1%) than in controls (21.0%) (odds ratio, 2.221; 95% confidence interval, 1.225–3.926; *p* = 0.0069; *Pc* = 0.048). The other *IL6* polymorphisms, rs1800797, rs2069829 and rs1800795, showed no differences between ANE cases and controls (Table 2).

**Table 2:** Genotype and allele frequencies: Comparison between ANE cases and control subjects.

Gene	SNP		Genotype			Allele		Genotype frequency		Allele frequency		Reference	SNP	*p*	*Pc*	*p*	*Pc*
***IL6***	**rs1800797**		**GG**	**GA**	**AA**	**G**	**A**				
	(−597 G > A)	ANE	31 (100%)	0 (0%)	0 (0%)	62 (100%)	0 (0%)	1.000	1.000	1.000	1.000
		Control	45 (100%)	0 (0%)	0 (0%)	90 (100%)	0 (0%)				
	**rs1800796**		**CC**	**CG**	**GG**	**C**	**G**				
	(−572 C > G)	ANE	11 (35.5%)	17 (54.8%)	3 (9.7%)	39 (62.9%)	23 (37.1%)	**0.021**	0.146	**0.006**	**0.048**
		Control	89 (62.2%)	48 (33.6%)	6 (4.2%)	226 (79.0%)	60 (21.0%)				
	**rs2069829**		**CC**	**CT**	**TT**	**C**	**T**				
	(−190 C > T)	ANE	31 (100%)	0 (0%)	0 (0%)	62 (100%)	0 (0%)	1.000	1.000	1.000	1.000
		Control	45 (100%)	0 (0%)	0 (0%)	90 (100%)	0 (0%)				
	**rs1800795**		**GG**	**GC**	**CC**	**G**	**C**				
	(−174 G > C)	ANE	31 (100%)	0 (0%)	0 (0%)	62 (100%)	0 (0%)	1.000	1.000	1.000	1.000
		Control	45 (100%)	0 (0%)	0 (0%)	90 (100%)	0 (0%)				
***IL10***	**rs1800896**		**AA**	**AG**	**GG**	**A**	**G**				
	(−1,082 A > G)	ANE	25 (80.6%)	5 (16.1%)	1 (3.2%)	55 (88.7%)	7 (11.3%)	0.159	1.000	0.053	0.371
		Control	163 (90.6%)	16 (8.9%)	1 (0.5%)	342 (95.0%)	18 (5.0%)				
	**rs1800871**		**TT**	**TC**	**CC**	**T**	**C**				
	(−819 T > C)	ANE	16 (51.6%)	8 (25.8%)	7 (22.6%)	40 (64.5%)	22 (35.5%)	**0.003**	**0.024**	0.976	1.000
		Control	84 (37.0%)	124 (54.6%)	19 (8.4%)	292 (64.3%)	162 (35.7%)				
	**rs1800872**		**AA**	**AC**	**CC**	**A**	**C**				
	(−627 A > C)	ANE	16 (51.6%)	8 (25.8%)	7 (22.6%)	40 (64.5%)	22 (35.5%)	**0.003**	**0.024**	0.976	1.000
		Control	84 (37.0%)	124 (54.6%)	19 (8.4%)	292 (64.3%)	162 (35.7%)				

Bold values of genotype or allele frequencies show statistical significance.

Genotyping of *IL10* polymorphisms confirmed that two of the three common variants in the *IL10* promotor region, rs1800871 and rs1800872, show a complete linkage, as reported previously (Schuurhof et al., 2011; Zhang et al., 2012), resulting in rs1800871/rs1800872 TA and CC haplotypes, and in TA/TA, TA/CC and CC/CC diplotypes. The frequency of CC/CC diplotype was significantly higher in ANE (22.6%) than in control (8.4%) (CC/CC versus TA/CC and TA/TA: odds ratio, 3.193; 95% confidence interval, 1.245–8.515; *p* = 0.0137). The other *IL10* polymorphism, rs1800896, showed no differences between ANE cases and controls (Table 2).

### 3.2. IL-6/IL-10 production and *IL6*/*IL10* genotypes

**Figure 1:**
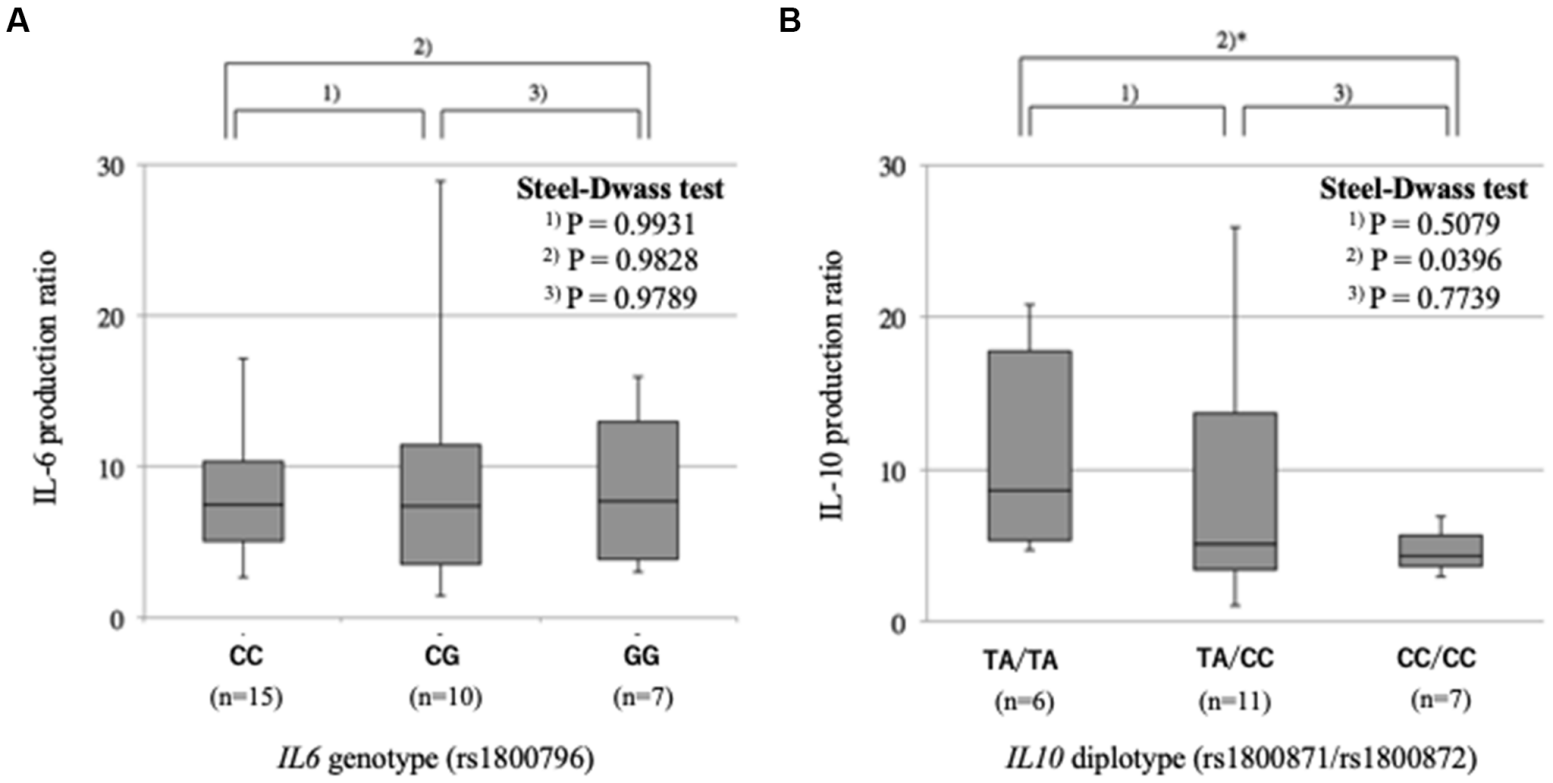
Effects of gene polymorphisms on cytokine production.IL-6 or IL-10 levels in cell supernatants were measured before and after stimulation of lymphoblasts by PMA. The ratios were compared between *IL6* genotypes or *IL10* diplotypes. **(A)** IL-6 production is comparable between the three *IL6* rs1800796 genotypes. **(B)** IL-10 production is low in IL10 rs1800871/rs1800872 CC/CC diplotype.

We examined whether the *IL6* rs1800796 and *IL10* rs1800871/rs1800872 polymorphisms are associated with the production of IL-6 and IL-10 proteins, respectively, by lymphoblasts upon PMA stimulation. The levels of IL-6 production were comparable among the three *IL6* rs1800796 genotypes, CC, CG and GG (*p* = 0.993 between CC and CG, *p* = 0.982 between CC and GG, and *p* = 0.979 between CG and GG) (Figure 1A). The levels of IL-10 production were different between the three *IL10* rs1800871/rs1800872 diplotypes, being high in TA/TA, moderate in TA/CC and low in CC/CC. The difference between TA/TA and CC/CC was statistically significant (*p* = 0.508 between TA/TA and TA/CC, *p* = 0.040 between TA/TA and CC/CC, and *p* = 0.774 between TA/CC and CC/CC) (Figure 1B).

## 4. Discussion

In this study, we first demonstrated by genetic analyses that the frequency of *IL6* rs1800796 G allele and that of *IL10* rs1800871/rs1800872 CC/CC diplotype were higher in ANE cases than in controls. However, statistical evidence provided by these analyses was not conclusive because the *p*-value for *IL6* rs1800796 was of borderline significance (*Pc* = 0.048), and because the genotypic frequency status of *IL10* rs1800871/rs1800872 in the control group was deviated from Hardy–Weinberg equilibrium. To further explore the pathogenetic significance of these polymorphisms, we next did functional analyses, and found that *IL10* rs1800871/rs1800872 CC/CC diplotype was associated with low production by lymphoblasts of IL-10 upon PMA stimulation, and that *IL6* rs1800796 allele G was not associated with IL-6 production upon PMA stimulation.

IL-6 is a proinflammatory cytokine secreted upon infection by various cells such as T lymphocytes, B lymphocytes, monocytes and endothelial cells. IL-6 plays a major role in immune responses such as differentiation/activation of T lymphocytes, enhancement of antibody production via proliferation of B lymphocytes, and induction of C reactive protein and other acute phase proteins in hepatocytes. In the promotor region of the *IL6* gene, there are four polymorphisms, rs1800797, rs1800796, rs2069829 and rs1800795. Previous studies have demonstrated their association with the onset, severity and/or response to treatment of various diseases including infectious, autoimmune, cardiovascular, gastrointestinal, neoplastic and neurologic disorders. Of these common variants, rs1800796 was identified by this study as a possible genetic risk factor of ANE. In chronic infectious diseases, rs1800796G allele, GG genotype or G-containing haplotype is reportedly associated with a decreased risk of tuberculosis (Zhang et al., 2012; Wang et al., 2017), increased risk of anti-tuberculosis drug-induced hepatotoxicity (Li et al., 2018), decreased prevalence of chronic human hepatitis B virus (HBV) infection (Zhang et al., 2015; Li et al., 2021), decreased chance of sustained viral response to antiviral therapy against chronic hepatitis C virus (HCV) infection (Yee et al., 2009; Sghaier et al., 2017b), and a decreased risk of progression from HCV infection to hepatocellular carcinoma (Sghaier et al., 2017b). In acute infectious diseases, rs1800796G allele is reportedly associated with an increased risk of sepsis in chronic renal disease (Panayides et al., 2015) and increased risk of post-traumatic osteomyelitis (Jiang et al., 2020). In acute viral infections that trigger ANE in most cases, information is currently limited. An Iranian study observed no effect of rs1800796 on the severity of COVID-19, whereas a Chinese study noted that a haplotype containing rs1800796G may be associated with an increased severity (Chen et al., 2021). No data are currently available as to influenza, the commonest antecedent infection of ANE.

IL-6 expression may be altered by *IL6* haplotypes/diplotypes consisting of multiple polymophisms. A previous study compared IL-6 production in the whole blood upon stimulation by lipopolysaccharide (LPS) between *IL6* diplotypes consisting of three polymorphisms, rs1800797, rs1800796 and rs1800795, and found that GGG/GGG diplotype (allele G for all the three including rs1800796) is associated with lower IL-6 production compared to the other diplotypes (Muller-Steinhardt et al., 2007). Other studies also observed an association of rs1800796G with a low production of IL-6 by CD14(+) monocytes in response to HBV core antigen stimulation (Zhang et al., 2015) and to *Mycobacterium tuberculosis* 19-kDa lipoprotein (Zhang et al., 2012). All these findings are evidence that rs1800796G is a low producer of IL-6 protein. However, another study found low levels of methylation in IL-6-related CpG sites, suggesting a high producer (Smallwood et al., 2008). In this study, we found no differences in IL-6 production between rs1800796 genotypes. The discrepancy among the studies may indicate that genetic polymorphisms in the promotor region influence *IL6* transcription not by a simple additive mechanism, but rather through complex interactions determined by haplotype (Terry et al., 2000). Whether rs1800796 and/or another polymorphism in linkage disequilibrium can alter IL-6 production in acute viral infections remains to be elucidated. The roles of IL-6 in the etiology and pathogenesis of ANE warrants vigorous investigation in view of the recent reports on the efficacy of an IL-6 antagonist, tocilizumab, in the treatment of ANE (Koh et al., 2019; Ho et al., 2023; Hosie et al., 2023).

IL-10 is an anti-inflammatory cytokine activated in an early phase of infection to suppress proinflammatory cytokines, thereby avoiding excessive immune responses and consequent tissue damages. IL-10 regulates immune function by suppressing production of interferon-γ by T lymphocytes and production of interleukin-1 (IL-1), IL-6, interleukin-2 (IL-2) and tumor necrosis factor α (TNFα) by macrophages (Sabat et al., 2010). In the promotor region of the *IL10* gene, there are three SNPs, rs1800896, rs1800871 and rs1800872, associated with the onset, severity, progression and/or outcome of various diseases including autoimmune, allergic, neoplastic, gastrointestinal and respiratory diseases. This study showed that a combination of two SNPs in complete linkage, rs1800871/rs1800872CC/CC diplotype, may be a genetic risk factor of ANE. In various infectious diseases, rs1800871C and/or rs1800872C is reportedly associated with the onset of chronic infections: a decreased susceptibility to tuberculosis (Yu et al., 2019; Chen and Ma, 2020), increased susceptibility to leprosy (Cardona-Castro et al., 2012; Dos Santos et al., 2021), decreased susceptibility to herpes zoster (Haanpää et al., 2002), and increased susceptibility to HBV infection (Ye et al., 2020). These alleles are also associated with the course, progression, response to therapy and outcome: a better outcome of leprosy (Alvarado-Arnez et al., 2015), low infection intensity of *Schistosoma mansoni* in schistosomiasis (Mewamba et al., 2023), reduced risk of cardiomyopathy in Chagas disease (Grijalva et al., 2022), reduced risk of anemia in newborns in Plasmodium falciparum malaria infection (Lokossou et al., 2013), increased risk of Epstein–Barr virus (EBV)-associated hemophagocytic lymphocytosis (Tang et al., 2021), reduced risk of breast cancer after EBV infection (He et al., 2012), increased risk of AIDS-related non-Hodgkin lymphoma (Wong et al., 2010), increased risk of progressive liver disease in chronic hepatitis B (Miyazoe et al., 2002), reduced chance of seroclearance after antiviral treatment of chronic hepatitis B (Rybicka et al., 2020), poor reduced disease severity of chronic hepatitis C (Świątek-Kościelna et al., 2017), and reduced risk of hepatocellular carcinoma in chronic hepatitis C (Aroucha et al., 2016; Sghaier et al., 2017a). For acute infections, s1800871C and/or rs1800872C is reportedly associated with a decreased risk of dengue infection (Eloisa Monroy-Muñoz et al., 2023) and dengue hemorrhagic fever (Alagarasu et al., 2015), decreased risk of asthma after bronchiolitis in infancy (Korppi et al., 2017), and reduced severity (requiring admission to intensive care unit) of sepsis (Montoya-Ruiz et al., 2016). In acute viral infections, these SNPs were reportedly associated with neither the prevalence of influenza (Mehrbod et al., 2021) nor the prevalence, severity and outcome of COVID-19 (Avendaño-Félix et al., 2021; Karcioglu Batur and Hekim, 2021; Abbood et al., 2023).

IL-10 expression may be altered by these *IL10* polymorphisms. Previous studies have demonstrated that *IL10* GCC haplotype (rs1800896G, rs1800871C and rs1800872C) is associated with a high IL-10 production by peripheral blood mononuclear cells (PBMCs) in response to LPS stimulation (Crawley et al., 1999; Edwards-Smith et al., 1999). Other studies also showed the association of rs1800872C and ACC haplotype (rs1800896A, rs1800871C and rs1800872C) with a high plasma level of IL-10 (Ge et al., 2015). By contrast, the present study showed an association of rs1800871/rs1800872 diplotype CC/CC with a low IL-10 production by lymphoblasts in response to PMA stimulation. The direction of alteration observed in this study is apparently opposite to that in previous studies, which may be explained by differences in cell types used, ethnicities, methods of stimulation, and other polymorphisms in high linkage. Taken together with the genotyping data, the results of this study suggest the possibility that the *IL10* rs1800871/rs1800872 polymorphism may predispose Japanese children by altering IL-10 production in the early phase of infection, which in turn leads to excessive immune responses, cytokine storm, then culminates in severe brain damage and multiorgan dysfunction.

This study had several limitations. First, the small sample size of ANE cases limited statistical power, precluding analyses of haplotypes consisting of three alleles. Due to the low incidence, fulminant clinical course and high mortality, it was very difficult to obtain a large number of ANE samples. However, to the best of our knowledge, our repository of genomic DNA is the only one in the world. Second, the control subjects, healthy Japanese adults, derived from three sources. Their validity as controls might have been affected by possible demographic differences among the three control groups and from the ANE group. Third, the use of lymphoblasts, not of PBMCs, in the cytokine production assay rendered the experimental condition quite different from the clinical situation of human viral infections. On the other hand, the experimental data were highly reproducible because of the homogeneity and stability of lymphoblast cell culture. Fourth, our results failed to account for the high incidence of ANE in Asia, since the frequencies if *IL6* rs1800796G and *IL10* rs1800781C are reportedly lower in Asians (<20 and 40%, respectively) than in Europeans (95 and 75%, respectively) (The 1000 Genomes Project Consortium, 2012; Yu et al., 2013).

In conclusion, we studied polymorphisms in the promotor region of the *IL6* and *IL10* gene, in an attempt to explore the roles of IL-6 and IL-10 as disease modifying factors of ANE. Genetic studies suggested possible associations of *IL6* rs1800796 and *IL10* rs1800781/rs1800782 with the onset of ANE. Functional analyses showed an altered expression of IL-10 by *IL10* rs1800781/rs1800782, but not that of IL-6 by *IL6* rs1800796. Taken together, the *IL10* rs1800781/rs1800782 polymorphism is suggested as a genetic risk factor of ANE, whereas the pathogenetic significance of *IL6* rs1800796 polymorphism remains to be elucidated.

## Data availability statement

The original contributions presented in the study are included in the article/supplementary material, further inquiries can be directed to the corresponding author.

## Ethics statement

The studies involving human participants were reviewed and approved by The Ethics Committee of the Graduate School of Medicine, the University of Tokyo. Written informed consent to participate in this study was provided by the participants’ legal guardian/next of kin.

## Author contributions

AH and MM designed the study and collected samples. NT, AO, and MM supervised the study. AH conducted the experiments and interpreted the data. AH and MM wrote the manuscript. All authors contributed to the article and approved the submitted version.

## Funding

This research was supported by Grants-in-Aid for Scientific Research, Nos. 18K15700 and 15H04842, from the Japan Society for the Promotion of Sciences, and a Grant-in-Aid for Policy Research on Intractable Diseases, No. 21FC1005, from the National Institute of Public Health, Japan.

## Conflict of interest

The authors declare that the research was conducted in the absence of any commercial or financial relationships that could be construed as a potential conflict of interest.

## Publisher’s note

All claims expressed in this article are solely those of the authors and do not necessarily represent those of their affiliated organizations, or those of the publisher, the editors and the reviewers. Any product that may be evaluated in this article, or claim that may be made by its manufacturer, is not guaranteed or endorsed by the publisher.
